# National Healthcare Safety Network… we need to talk…

**DOI:** 10.1017/ash.2025.10157

**Published:** 2025-10-01

**Authors:** Misti Ellsworth, Luis Zeichner Ostrosky, Bela Patel, Robert Yetman, Phillip Chang

**Affiliations:** 1 Division of Pediatric Infectious Diseases, McGovern Medical School at UTHealth Houston; Children’s Memorial Hermann Hospital, Houston, TX, USA; 2 Division of Infectious Diseases, Department of Internal Medicine, McGovern Medical School at UTHealth Houston; Memorial Hermann Texas Medical Center, Houston, TX, USA; 3 Division of Critical Care Medicine, McGovern Medical School at UTHealth Houston; Memorial Hermann Health System, Houston, TX, USA; 4 Department of Pediatrics, McGovern Medical School at UTHealth Houston; Children’s Memorial Hermann Hospital, Houston, TX, USA; 5 Memorial Hermann Health System, Houston, TX, USA

**Dear Editor,**

We are writing to highlight a critical issue observed in our recent review of bloodstream infection (BSI) classifications at a tertiary care center, which we believe has broader relevance for infection prevention programs: the increasing misattribution of multiple causes of bacteremia as central line-associated bloodstream infections (CLABSIs) under the current National Healthcare Safety Network (NHSN) definitions.

Over the past decade, the U.S. healthcare system has made significant strides in reducing CLABSI rates. However, recent data suggest a deceleration in progress.^1^ One possible reason for this stagnation lies in the evolution of NHSN’s definitions for secondary BSI attribution. By adding rigid symptom, culture, and imaging requirements, these definitions have improved the specificity of site-specific infection criteria. However, this has made attribution to those sites more difficult.

In our review of 282 CLABSI cases coded by a highly trained central team of infection preventionists—demonstrating a greater than 90% inter-rater agreement—strictly applying NHSN definitions from 2019 to 2023 at a large tertiary hospital, 43% of cases potentially had an alternative primary infection source when reviewed by infectious diseases-trained physicians.^2^ The most common were pneumonia (27%) and gastrointestinal infections (23%), followed by skin/soft tissue infections, vascular infections, endocarditis, and bacterial translocation. Many potential sources were not captured under NHSN criteria due to absent confirmatory imaging or matching site cultures within the strict infection window or repeat infection time frame. For example, in several mechanically ventilated patients with new infiltrates, purulent secretions, and leukocytosis, pneumonia could not be assigned because confirmatory chest imaging was not obtained in the infection window period as imaging was not clinically relevant. Similarly, patients with echocardiographic evidence of vegetations and compatible bacteremia did not meet the endocarditis definition because of rigid organism- and timing-based restrictions.

We also recognize that misattribution occurs in both directions—true line infections may be assigned to other sources, such as pneumonia or urinary tract infection, under the current definitions—further contributing to the misalignment between surveillance data and prevention priorities.

These attribution challenges are magnified in certain populations: pediatric patients, solid organ and hematopoietic transplant recipients, patients with mechanical circulatory support devices (eg, balloon pumps), and those who are profoundly immunosuppressed. In these groups, diagnostic procedures may be limited, organisms may not behave according to typical pathogenicity assumptions, and symptoms may be atypical, further complicating adherence to rigid surveillance criteria.

We believe there are several opportunities for NHSN to refine the CLABSI definition and related attribution rules to better align with clinical realities:

- Consider a tiered approach that incorporates elements of the IDSA clinical definition for catheter-related BSI, especially in complex or high-acuity patients.
- Allow documented clinician judgment as a tiebreaker in cases where strict imaging or microbiologic criteria cannot be obtained for plausible alternative sources.
- Revisit infection window period and repeat infection time frame rules for immunocompromised and critically ill populations.
- Reassess the excluded organism lists and consider expanding the mucosal barrier injury/commensal organism category, particularly for low-likelihood central line pathogens in specific populations.
- Pilot test modified definitions with real-world case review to assess impact on attribution accuracy.

Finally, the imminent transition to digital clinical quality measures, including hospital-onset bacteremia (HOB), will likely exacerbate these challenges. Current HOB proposals lack risk adjustment and do not account for secondary sources, risking inflated rates in high-acuity centers and diverting prevention resources from true line infections. We recommend that HOB definitions incorporate structured fields for clinician-documented secondary sources, enabling nuanced attribution while preserving the feasibility of automated abstraction.

Inaccurate attribution has meaningful downstream consequences: quality metrics may lose credibility, prevention efforts may be misdirected, and institutions committed to improvement may face undue penalties. Surveillance criteria must evolve alongside clinical practice to ensure that reported metrics reflect true preventable events and guide meaningful quality improvement.

Thank you for considering this perspective. We hope these suggestions contribute to the ongoing dialogue on optimizing infection surveillance for the benefit of patient safety.

Sincerely,

**Luis Ostrosky, MD, FACP, FIDSA, FSHEA, FECMM, CMQ**

Professor of Medicine and Memorial Hermann Endowed Chair Vice Chairman for Healthcare Quality, Department of Internal Medicine Chief, Division of Infectious Diseases McGovern Medical School Medical Director of Epidemiology - Memorial Hermann Texas Medical Center

**Misti Ellsworth, DO**

Associate Professor, Pediatrics Infection Prevention - Children’s Memorial Hermann Hospital Division of Pediatric Infectious Diseases McGovern Medical School at UTHealth Houston

**Bela Patel, MD, FCCP, FCCM, ATSF**

Professor, Graham Distinguished University Chair Vice Dean, Healthcare Quality Director, Division of Critical Care Medicine McGovern Medical School at UTHealth Houston Chief Medical Officer—Memorial Hermann

**Robert Yetman, MD**

Professor of Pediatrics Vice-Chair, Clinical Operations McGovern Medical School at UTHealth Houston Chief Medical Officer—Children’s Memorial Hermann Hospital

**Phillip K. Chang, MD, MBA, FACS**

Senior Vice President, Chief Medical and Quality Officer Memorial Hermann Health System
